# Multi-Response Optimisation of Automotive Door Using Grey Relational Analysis with Entropy Weights

**DOI:** 10.3390/ma15155339

**Published:** 2022-08-03

**Authors:** Hao Chen, Chihua Lu, Zhien Liu, Cunrui Shen, Menglei Sun

**Affiliations:** 1 Hubei Key Laboratory of Advanced Technology for Automotive Components, Wuhan University of Technology, Wuhan 430070, China; chenhao2014@whut.edu.cn (H.C.); chlu@whut.edu.cn (C.L.); scray@whut.edu.cn (C.S.); sun_ml@whut.edu.cn (M.S.); 2 Foshan Xianhu Laboratory of the Advanced Energy Science and Technology Guangdong Laboratory, Xianhu Hydrogen Valley, Foshan 528200, China

**Keywords:** automotive door, lightweight, Taguchi, grey relational analysis, entropy method, multi-response optimisation

## Abstract

Tail-welded blanks (TWBs) are widely used in automotive bodies to improve the structural performance and reduce weight. The stiffness and modal lightweight design optimisation of TWBs for automotive doors was performed in this study. The finite element model was validated through physical experiments. An L27 (3^12^) Taguchi orthogonal array was used to collect the sample points. The multi-objective optimisation problem was transformed into a single-objective optimisation problem based on the grey relational degree. The optimal combination of structural design parameters was obtained for a tail-welded door using the proposed method, and the weight of the door structure was reduced by 2.83 kg. The proposed optimisation method has fewer iterations and a lower computational cost, enabling the design of lightweight TWBs.

## 1. Introduction

Lightweight materials have become a popular research topic in the automotive industry in an effort to save energy and reduce exhaust emissions. There are two primary means of reducing automobile weight: lightweight materials [1,2,3] and lightweight structures [4,5,6]. Lightweight structures achieve weight reduction through the use of new structures. Lightweight materials include aluminium, magnesium alloy, and other materials with lower density, replacing traditional iron and steel materials in thin-walled panels to achieve weight reduction. Most thin-walled parts are stamped and welded from a single piece of material. The stamping die is large, and the production cost is high. When the strength and stiffness requirements of the door are met, there are redundant materials, increasing the weight of the door, fuel consumption, and emissions.

The door assembly is an important part of an automobile, and producing a lighter door structure that meets stiffness and noise, vibration, and harshness (NVH) performance needs is a key requirement. To reduce the weight of automotive doors, TWBs have become popular in automotive engineering [7,8,9,10]. Li et al. [11] proposed a lightweight automotive door design with a TWB structure in several load cases. Sun et al. proposed the compromise programming approach coupled with the mean frequency method to handle the multi-objective optimisation involving vehicle door stiffness and natural frequency criteria for multiple load cases. Zhao et al. [12] developed an effective approach for the robust design optimisation of car-door structures with spatially varied material uncertainties. Previous door optimisation designs only considered a single material, making it difficult to meet stiffness and dynamic requirements, and the utilisation efficiency of the door material was not maximised.

Traditional discrete variable design optimisation methods such as the genetic algorithm and the particle swarm optimisation algorithm are expensive in terms of calculation costs for the automotive body system. The Taguchi method is used as an efficient and frugal design method that is scientific and practical in exploring the optimal state. It is a special design method which reduces the number of tests by utilizing mathematical fundamentals [13,14,15]. Liu et al. [16] established a multi-body dynamic model of a suspended monorail vehicle. The Taguchi method was used to determine the optimal combination of suspension parameters, which improved the lateral and vertical running stability of the vehicle. Shrestha et al. [17] studied the relationship between the print parameters and transverse rupture strength of sintered 316L stainless-steel using the Taguchi method and determined the best additive manufacturing parameters to improve the transverse rupture strength. However, the structural optimisation of a door design must consider the stiffness, NVH, and weight of the door, indicating a multi-objective optimisation problem. A single Taguchi analysis is only applicable to single-objective optimisation, greatly limiting its application.

Grey relational analysis (GRA) with entropy weights can solve multi-objective optimisation problems with multiple criteria, and its application in multi-objective problems has gained popularity [18,19,20]. She et al. [21] optimised the bending performance of optical fibres using grey relational analysis and found that the bending loss was reduced by an order of magnitude. Dabwan et al. [22] conducted experimental research on incremental sheet forming, using the grey relational method with entropy weights to determine the optimum process variables for single-point incremental forming. There is still great capacity to use multi-objective discrete optimisations in numerical studies and integrate them with design processes of complex structures such as automotive bodies.

Most researchers use GRA for multi-objective optimisation without considering the robustness of the system and combine it with Taguchi analysis. In terms of door design variables, the discreteness of panel material types and the panel thicknesses are the most important features. However, few studies have focused on the structural stiffness of doors and NVH optimisation design considering discrete variables, and fewer studies have adopted GRA with entropy weights.

**Figure 1:**
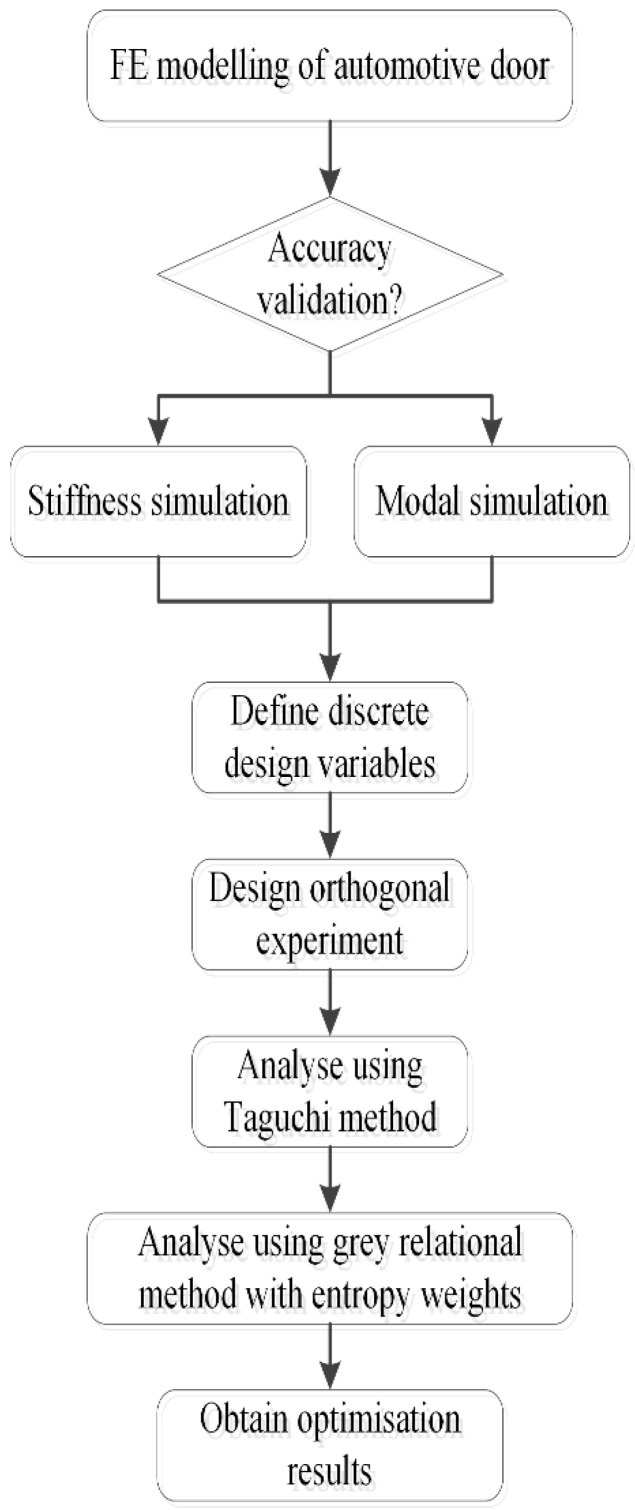
Flowchart of the lightweight optimisation method.

In this study, a finite element model of an automotive door was established. Orthogonal experiments were conducted using the Taguchi method by changing the panel thickness and panel material design variable combinations. Grey relational analysis and entropy weight were used to optimise the automotive door panel design, and the multi-objective optimisation problem was transformed into a single-objective optimisation problem. The optimised results, the significant influencing factors, and the optimal level combination were determined. The results show that the optimised structure reduces the weight to some extent, and the door performance meets the baseline requirements. These findings provide guidance for the design of similar structures. Figure 1 shows a flowchart of the proposed lightweight optimisation method.

## 2. Methodology

### 2.1. Taguchi Method

The Taguchi method is an optimisation design method based on experimental design, and the optimisation process was performed in accordance with experimental results. Selection of experimental parameters is the top priority in all optimisation studies [23]. Equation (1) is often used to obtain the signal-to-noise ratio (S/N ratio) of ‘the larger the better’ response, while Equation (2) is used to obtain the S/N ratio of ‘the smaller the better’ response:

(1) $$\eta_{Larger}=-10\mathrm{lg}(\frac{1}{m}\sum_{i=1}^{m}\frac{1}{y_{ij}^{2}})$$

(2) $$\eta_{Smaller}=-10\mathrm{lg}(\frac{1}{m}\sum_{i=1}^{m}y_{ij}^{2})$$

### 2.2. Grey Relational Analysis with Entropy Weights

To optimise the door structure, the influence of the variables on the results must be understood. Grey relational theory effectively measures the influence of different variables. With the experimental data, grey relational theory determines the variables with the greatest influence. In addition to considering the influence of each variable on the objective function separately, grey relational theory can also consider the mutual influence of multiple variables [24,25].

As the dimensions and orders of magnitude of each evaluation index are different, each parameter must be normalised to eliminate the impact of different dimensions on the results. The normalisation method is usually described as follows:

For the larger the better response:

(3) $$x_{ij}^{*}=\frac{x_{ij}-\min x_{j}}{\max x_{j}-\min x_{j}}\ldots i=1,2,\ldots ,m\ldots j=1,2,\ldots ,n$$

For the smaller the better response:

(4) $$x_{ij}^{*}=\frac{\max x_{j}-x_{ij}}{\max x_{j}-\min x_{j}}\ldots i=1,2,\ldots ,m\ldots j=1,2,\ldots ,n$$

where $x_{ij}$ and $x_{ij}^{*}$ are the simulation and normalised values for the *j*^th^ response in the *i*^th^ trial, respectively, $\max x_{j}$ is the maximum value for the *j*^th^ response in all trials, $\min x_{j}$ is the minimum value for the *j*^th^ response in all trials, *m* is the number of trials, and *n* is the number of response indicators.

The normalised S/N ratio reference sequences and comparison sequences are used to calculate the grey relational coefficient (GRC) of the S/N ratio for each quality characteristic:

(5) $$\xi_{ij}=\frac{\underset{i}{\max}\underset{j}{\max}\Delta_{ij}+\rho \underset{i}{\min}\underset{j}{\min}\Delta_{ij}}{\Delta_{ij}+\rho \underset{i}{\min}\underset{j}{\min}\Delta_{ij}}$$

where $\xi_{ij}$ is the correlation coefficient of the one-to-one correspondence between the comparison sequence and the reference sequence for the new data of the *j*^th^ response in the *i*^th^ trial in the grey relational analysis of influencing factors, $x_{ij}^{\prime }$ is the comparison sequence, $\Delta_{ij}=\left|x_{ij}^{*}-x_{ij}^{\prime }\right|$ is the absolute difference of the *j*^th^ response in the *i*^th^ trial, and ρ is the grey relational resolution coefficient, whose value reflects the correlation integrity of each factor influencing the target value; generally, ρ=0.5.

To improve the evaluation accuracy of the grey relational analysis of factors influencing the target response value, the average correlation coefficient between each indicator factor in the new comparison sequence and the reference sequence is calculated as the grey relational degree:

(6) $$\gamma_{ij}=\frac{1}{n}\sum_{j=1}^{n}\xi_{ij}$$

The weighted sum of the grey relational coefficients is the grey relational degree, calculated as:

(7) $$\gamma_{ij}=\sum_{j=1}^{n}\beta_{j}\xi_{ij};\ldots \ldots \sum_{j=1}^{n}\beta_{j}=1$$

where $\beta_{j}$ is the weight value of the *j*^th^ response variable.

With the different roles and influences of each response indicator, different weights must be assigned according to the importance of each indicator. The entropy weight method was used to assign weights to the target values.

The entropy weight method determines an objective weight according to the change in the response. With a greater difference in response values, more information is provided, and a greater weight is assigned [26,27]. The weight calculation method based on the entropy value is as follows:

(1) Determine the geometric projection, $P_{ij}$, of each response:

(8) $$P_{ij}=\frac{1+x_{ij}^{*}}{\sum_{i=1}^{m}\left(1+x_{ij}^{*}\right)}$$

(2) Calculate the entropy, $E_{j}$:

(9) $$E_{j}=-\frac{1}{\mathrm{In}m}\sum_{i=1}^{m}P_{ij}\mathrm{In}P_{ij}$$

(3) Calculate the weight coefficient, $\omega_{j}$:

(10) $$\omega_{j}=\frac{1-E_{j}}{n-\sum_{j=1}^{n}E_{j}}$$

The weight coefficient reflects the amount of information in the index. An evaluation index may have different objective weights for different objects.

## 3. Finite Element Modelling and Experiment Validation for Automotive Door

### 3.1. Finite Element Modelling

The finite element model was pre-processed using HYPERWORKS, and the model was computed using MSC.NASTRAN. The automotive door comprises thin-walled parts (including inner and outer panels, support panels, interior panels, and glass), meshed using shell elements with three or four nodes. To prevent the model stiffness from becoming inaccurately large, the number of three-node shell elements was restricted to not more than 3% of the elements in the finite element model. The automotive door structure was high-strength steel and glass. Spot welding was used to connect the door parts, simulated with the element ACM2 (six-sided solid element and interpolation constraint element). The bonding material was adhesive. The material properties are shown in Table 1. There were 34,186 elements and 981 3-node shell elements (2.87%) in the automotive door.

### 3.2. Experiment Validation

The automotive door must have sufficient stiffness and vibration resistance to ensure safety and comfort. To meet the energy savings and emission reduction requirements, the door must be lightweight.

**Figure 2:**
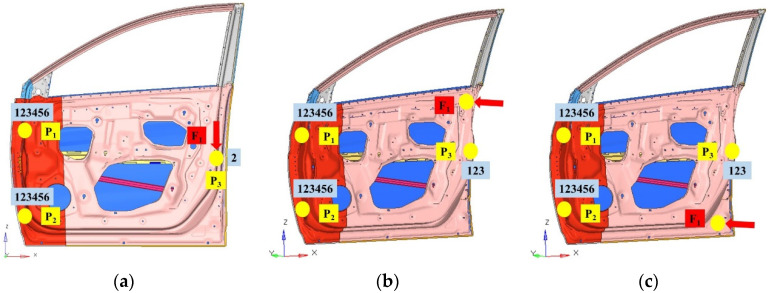
Loading and boundary conditions for stiffness analysis of the automotive door structure. (**a**) Vertical sag, (**b**) upper lateral, and (**c**) lower lateral.

**Figure 3:**
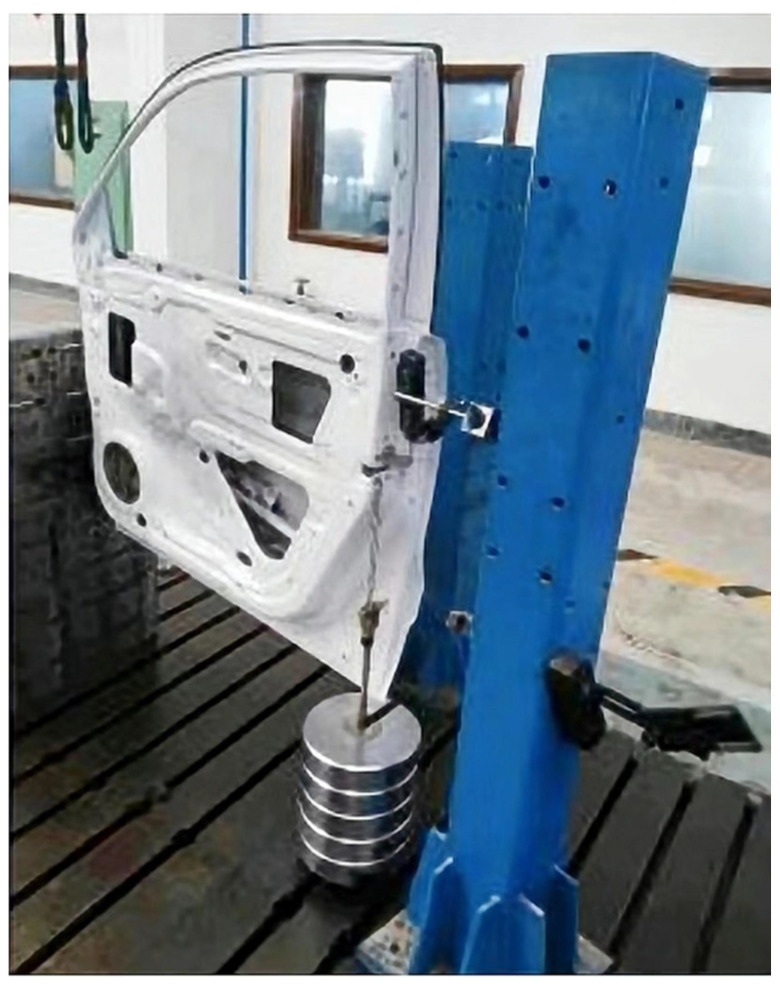
Experimental test of stiffness for the automotive door.

Several indicators can be used to measure the stiffness of the door: the vertical sag stiffness, upper lateral stiffness, and lower lateral stiffness are important [28]. Three load cases are presented in Figure 2. In condition 1: vertical sag case, as shown in Figure 2a, there are six degrees of freedom at the connection point between the hinge and the body (points P1 and P2) being constrained, and two degrees of freedom in the translational direction of the door latch (point P3) along the y-direction (transverse direction of body) being constrained. A force of 900 N in the direction of gravity was applied at point P3. In condition 2: upper lateral case, as shown in Figure 2b, there are 6 directional degrees of freedom, including 3 translational and 3 rotational degrees of freedom at the connection point between points P1 and P2 being constrained, and 3 translational degrees at point P3 being constrained. A 900 N force along with the y-axis was applied 5 mm below the edge line of the window frame in the upper left corner of the door inner panel. In condition 3: lower lateral case, as shown in Figure 2c, the lateral stiffness constraint conditions under the door are the same as those for upper lateral stiffness, but the applied load is different. A 900 N directional nodal force with two degrees of freedom was applied at the centre of the lower left corner of the inner panel of the door. The modal analysis of an automotive door considers its free mode. The first-order free mode of the door must meet certain requirements to prevent coupling resonance with the lower-order mode of the automotive body. The experimental setup is shown in Figure 3.

It is observed in Table 2 that the established finite element model can successfully predict the static and dynamic performance of the door structure with high precision and can be used for subsequent parameter analysis.

## 4. Multi-Objective Optimisation of Automotive Door

### 4.1. Design Variables

**Figure 4:**
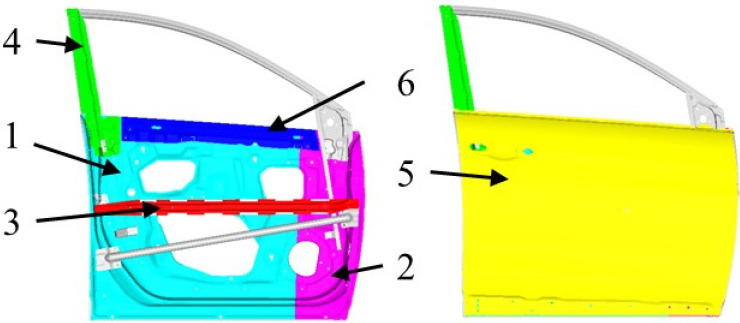
Schematic of door parts: 1: right inner panel, 2: left inner panel, 3: middle reinforcement of inner panel, 4: vertical belt reinforcement, 5: outer panel, and 6: transverse belt reinforcement.

The optimisation object is composed of six parts with different thicknesses and three parts with different material properties. The material types and thicknesses of each component were considered as independent discrete variables and divided into three levels for selection. The right inner panel, left inner panel, middle reinforcement of the inner panel, vertical belt reinforcement, outer panel, and transverse belt reinforcement have significant influences on the dynamic performance of the door, and are regarded as the optimisation objects, as shown in Figure 4. Three isotropic homogeneous materials were considered in this study: high-strength steel M1 (DP500), aluminium alloy M2 (ADC12), and magnesium alloy M3 (AM60). Their material properties are presented in Table 3. The range of each design variable is presented in Table 4.

After determining the design variables, an orthogonal experiment with 12 factors and 3 levels was designed, an L27 (3^12^) orthogonal array was selected, and values were assigned to the 12 design variables.

### 4.2. Multi-Objective Optimisation Model

In this study, the bending stiffness, first-order bending mode, and first-order torsional mode were considered constraints, and the values of the constraints were no less than 95% of the initial value. The panel thickness of six parts was taken as a design variable to obtain the mathematical model of multi-objective optimisation:

(11) min:M s.t. f≥f0 s.t. dsag ≤dsag 0 s.t. dupper≤dupper0 s.t. dlower ≤dlower 0

where $d_{sag}^{0}$ ($d_{sag}^{0}=1.64\ldots \mathrm{mm}$), $d_{upper}^{0}$($d_{upper}^{0}=1.03\ldots \mathrm{mm}$), and $d_{lower}^{0}$ ($d_{lower}^{0}=13.28\ldots \mathrm{mm}$) are the initial values of the sag stiffness displacement, upper lateral stiffness displacement, and lower lateral stiffness displacement, respectively. $f^{0}$($f^{0}=42\ldots \mathrm{Hz}$) is the low limit value of the first-order natural frequency.

### 4.3. Analysis of S/N Ratios

According to the quality characteristics, the results for multiple responses and the corresponding S/N ratios were calculated for the first-order natural frequency, the upper lateral stiffness displacement, the lower lateral stiffness displacement, and the mass, using Equations (1) and (2), and the results are shown in Table 5.

**Figure 5:**
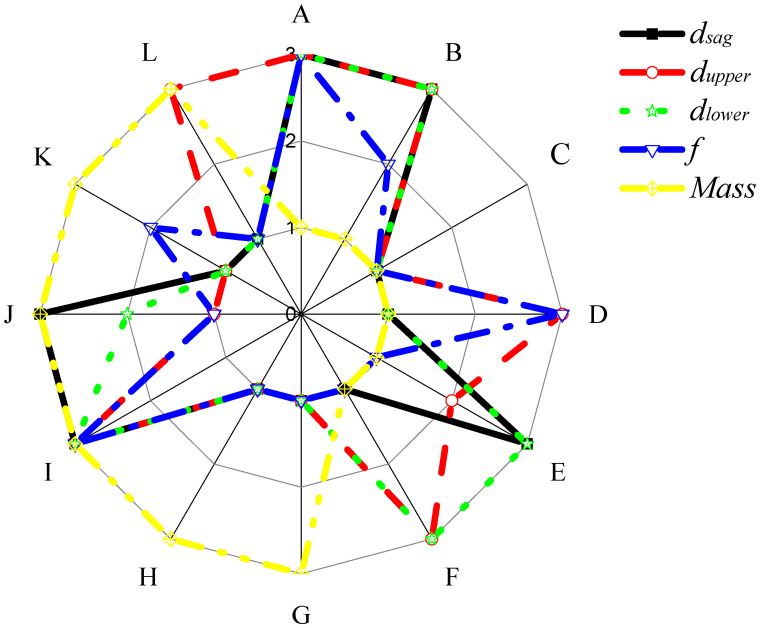
Single-objective optimisation for each response.

A level with a large S/N ratio is the optimal parameter level. Figure 5 shows the optimal horizontal combination of parameters in a single response. The best combination for *d*_*sag*_ is A_3_B_3_C_1_D_1_E_3_F_1_G_1_H_1_I_3_J_3_K_1_L_1_, the best combination for *d*_*upper*_ is A_3_B_3_C_1_D_3_E_2_F_3_G_1_H_1_I_3_J_1_K_1_L_3_, the best combination for *d*_*lower*_ is A_3_B_3_C_1_D_1_E_3_F_3_G_1_H_1_I_3_J_2_K_1_L_1_, the best combination for the first-order natural frequency, *f,* is A_3_B_2_C_1_D_3_E_1_F_1_G_1_H_1_I_3_J_1_K_2_L_1_, and the best combination for the mass is A_1_B_1_C_1_D_1_E_1_F_1_G_3_H_3_I_3_J_3_K_3_L_3_. The best combination for the mass is the material grade with the minimum thickness and density, which is consistent with the actual conditions. According to the analysis, the optimal parameter combinations are different for different responses. Multi-objective optimisation is required to meet the objectives of minimum mass, maximum stiffness, and maximum modal frequency.

### 4.4. Grey Relational Analysis

Using grey relational analysis, performance indicators of an automotive door can be transformed into a grey relational degree for comparative analysis to determine the optimal scheme.

Before grey relational analysis, the calculated S/N ratios for each response value were normalised to eliminate the influence of the dimension on the analysis. The experimental results were normalised and scaled to [0, 1], and the normalised results for each response value were calculated according to Equations (3) and (4) and are shown in Table 6. A larger normalised value indicates better performance, and a normalised value of 1 indicates the best performance.

The grey relational coefficient and the mean grey relational degree were calculated according to Equations (5)–(7), and the results are presented in Table 7. As the grey relational degree increases, the factors are closer to the optimal combination. The mean value of the grey relational degree also indicates the optimal parameter combination index. When the mean value of the grey relational degree corresponding to a factor level is the largest, its corresponding performance response is the best.

It was observed that H had the greatest influence on *d*_*sag*_ (Δ=0.4469), followed by B (Δ=0.1900), from the ranking of the mean grey relational degrees of different factor levels. Of the factors that affected *d*_*upper*_, the effects of G and A were significant, with ranges of Δ=0.3240 and Δ=0.2705, respectively. Of the factors that affected *d*_*lower*_, the effects of G and A were significant, with ranges of Δ=0.3262 and Δ=0.2298, respectively. A had the greatest influence on the first-order natural frequency, *f*, (Δ=0.2680), followed by G (Δ=0.1138). In terms of weight reduction, K had the greatest influence (Δ=0.2431), followed by G (Δ=0.1972), indicating that the material properties had a greater influence than the panel thickness.

The entropy weight method is an objective method of value assignment that measures the relative importance of the indicators according to the uncertainty of each indicator. According to the grey relational coefficients in Equations (8)–(10), and Table 7, the weight values for *d*_*sag*_, *d*_*upper*_, *d*_*lower*_, *f*, and mass were 0.1744, 0.1696, 0.2087, 0.2360, and 0.2113, respectively, representing their importance to the target value. The optimal solution is determined according to the grey relational degree.

**Figure 6:**
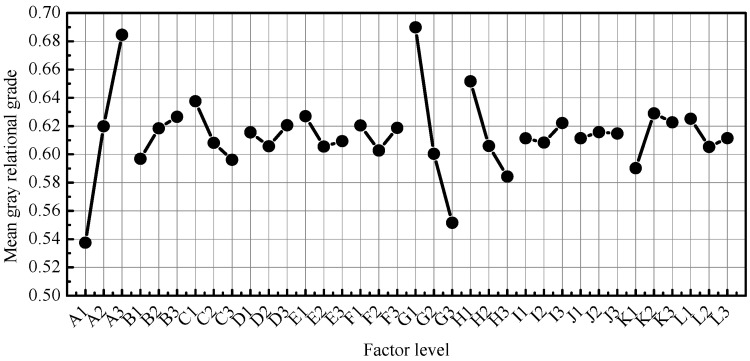
Main effects of factor levels.

The influence of the door design parameters is shown in Figure 6. The maximum mean values of the grey relational degree for A, B, C, D, E, F, G, H, I, J, K, and L can be expressed as A_3_, B_3_, C_1_, D_3_, E_1_, F_1_, G_1_, H_1_, I_3_, J_2_, K_2_, and L1, respectively. Thus, the best combination of door structural design parameters was A_3_B_3_C_1_D_3_E_1_F_1_G_1_H_1_I_3_J_2_K_2_L_1_. The thicknesses of the right inner panel, left inner panel, middle reinforcement panel of the inner panel, window frame vertical reinforcement panel, outer panel, and window frame horizontal reinforcement panel were 0.9, 1.6, 0.5, 1.0, 0.5, and 0.6 mm, respectively. The right inner panel, left inner panel, and window frame horizontal reinforcement panel were high-strength steel, the window frame vertical reinforcement panel and the outer panel were aluminium alloy, and the middle reinforcement panel of the inner panel was magnesium alloy.

The optimal combination of parameters was assigned to the finite element model for simulation analysis, and the final structural weight and dynamic performance parameters of the door were calculated, as shown in Table 8. With the lightweight design, the weight of the door structure was reduced by 2.83 kg. The performance of the door increased and decreased but met all baseline design requirements.

## 5. Conclusions

In this study, the lightweight TWB structure of an automotive door was considered as the research object. Through finite element analysis, the dynamic performance and lightweight indicators of the automotive door were obtained, and the accuracy of the finite element model was verified through experiments. The main conclusions are presented as follows.

(1) A multi-objective discrete optimisation design was successfully developed through grey relational analysis of the S/N ratio. With only 27 iterations, this method is a discrete optimisation design with low computational costs and cost-effectiveness. Thus, it is more suitable than conventional methods for complex optimisation problems.
(2) The grey relational method is feasible for optimisation. The Taguchi method and grey relational method were used to analyse the results. The number of experiments was reduced, and the influence of each parameter on the results was measured. The entropy weight method was used to obtain the weight value of each target response and determine the optimal combination of structural parameters. Grey relational analysis with entropy weights can significantly improve the comprehensive structure performance.
(3) The optimisation results indicated that the weight of the door structure was reduced by 2.83 kg. The performance of the door increased and decreased but met all baseline design requirements. This method can effectively realise lightweight door design and has a high value in engineering applications.

## Figures and Tables

**Table 1:** Material properties of finite element modelling.

Material	Young’s Modulus (MPa)	Mass Density (kg/m^3^)	Poisson’s Ratio
High-strength steel	2.1 × 10^5^	7.85 × 10^3^	0.3
Glass	6.9 × 10^4^	2.5 × 10^3^	0.3
Adhesive	50	1.2 × 10^3^	0.49

**Table 2:** Comparison of FE simulations and experimental test.

Parameter	Simulation	Experiment	Error (%)
Mass, *M* (kg)	27.71	28.03	−1.14
Natural frequency, *f* (Hz)	42.68	40.92	4.30
Vertical sag, *d*_*sag*_ (mm)	1.64	1.67	−1.80
Upper lateral, *d*_*upper*_ (mm)	1.03	1.06	−2.83
Lower lateral, *d*_*lower*_ (mm)	13.28	13.36	−0.60

**Table 3:** Material properties of door structure.

ID	Material	Elastic Modulus (GPa)	Poisson’s Ratio	Density (kg/m^3^)
1	Steel	210	0.30	7850
2	Aluminium	72	0.30	2770
3	Magnesium	45	0.33	1740

**Table 4:** Discrete design variables and corresponding values.

Design Variable	Value Range		
	Level 1	Level 2	Level 3
A (mm)	0.5	0.7	0.9
B (mm)	1.2	1.4	1.6
C (mm)	0.5	0.6	0.8
D (mm)	0.6	0.8	1.0
E (mm)	0.5	0.7	0.9
F (mm)	0.6	0.8	1.0
G	DP500	ADC12	AM60
H	DP500	ADC12	AM60
I	DP500	ADC12	AM60
J	DP500	ADC12	AM60
K	DP500	ADC12	AM60
L	DP500	ADC12	AM60

**Table 5:** Simulation results and corresponding S/N ratios.

No.	*d_sag_* (mm)	S/N	*d_upper_* (mm)	S/N	*d_lower_* (mm)	S/N	*f* (Hz)	S/N	*M* (kg)	S/N
1	2.411	−7.667	1.720	−4.858	1.06	−16.106	37.74	31.598	24.49	−27.833
2	6.571	−16.393	4.006	−12.213	2.33	−22.941	29.83	29.434	19.08	−25.647
3	10.070	−20.106	5.786	−15.409	3.22	−25.744	26.65	28.461	18.01	−25.142
4	4.786	−13.594	2.119	−6.628	1.49	−18.991	37.44	31.462	19.98	−26.032
5	6.771	−16.661	3.859	−11.890	2.11	−22.090	29.61	29.365	22.05	−26.966
6	2.303	−7.267	4.316	−12.884	2.44	−23.357	27.15	28.611	21.70	−26.770
7	5.115	−14.196	2.080	−6.484	1.39	−18.421	37.21	31.405	20.13	−26.092
8	1.922	−5.636	3.075	−9.930	2.51	−23.627	30.49	29.623	21.50	−26.661
9	3.637	−11.256	4.576	−13.388	2.41	−23.262	26.53	28.369	23.80	−27.649
10	2.672	−8.568	3.079	−9.939	1.86	−20.958	32.44	30.156	22.16	−26.976
11	6.107	−15.753	1.127	−1.154	0.92	−14.776	39.55	31.946	22.20	−26.943
12	9.520	−19.616	2.362	−7.615	1.70	−20.178	37.19	31.335	19.30	−25.739
13	5.049	−14.064	3.088	−9.940	2.30	−22.770	32.89	30.278	18.70	−25.456
14	6.331	−16.075	1.318	−2.510	0.82	−13.844	37.02	31.360	24.92	−28.003
15	1.957	−5.840	2.514	−8.152	1.41	−18.540	36.24	31.111	21.73	−26.782
16	5.394	−14.661	3.277	−10.464	2.28	−22.725	32.65	30.214	18.53	−25.383
17	1.554	−3.768	1.335	−2.399	0.89	−14.446	41.41	32.304	23.81	−27.551
18	3.264	−10.315	1.959	−6.009	1.31	−17.953	37.54	31.417	24.28	−27.807
19	2.427	−7.725	1.628	−4.373	1.10	−16.428	37.62	31.601	22.99	−27.289
20	6.329	−16.065	2.398	−7.737	1.65	−19.940	37.62	31.427	19.67	−25.915
21	9.229	−19.345	1.121	−0.966	0.80	−13.580	38.94	31.810	22.62	−27.095
22	4.780	−13.585	1.903	−5.693	1.49	−18.813	41.73	32.424	19.27	−25.709
23	6.554	−16.377	1.844	−5.485	1.49	−19.150	38.99	31.742	22.34	−27.065
24	1.702	−4.618	0.906	0.841	0.60	−11.138	43.36	32.725	25.50	−28.156
25	5.106	−14.180	1.687	−4.657	1.35	−18.158	40.96	32.332	19.82	−25.970
26	1.771	−4.913	2.337	−7.510	1.56	−19.372	38.06	31.536	20.82	−26.386
27	3.022	−9.641	0.986	0.087	0.57	−10.601	38.03	31.595	27.52	−28.876

**Table 6:** Normalisation of S/N ratios (NOR) and grey relational coefficient (GRC) for each performance characteristic.

No.	*d*_*sag*_		*d*_*upper*_		*d*_*lower*_		*f*		*Mass*	
	NOR	GRC	NOR	GRC	NOR	GRC	NOR	GRC	NOR	GRC
1	0.239	0.396	0.351	0.435	0.364	0.440	0.741	0.659	0.721	0.641
2	0.773	0.687	0.803	0.718	0.815	0.730	0.245	0.398	0.135	0.366
3	1.000	1.000	1.000	1.000	1.000	1.000	0.021	0.338	0.000	0.333
4	0.601	0.556	0.460	0.481	0.554	0.529	0.710	0.633	0.238	0.396
5	0.789	0.703	0.783	0.698	0.759	0.674	0.229	0.393	0.489	0.494
6	0.214	0.389	0.845	0.763	0.842	0.760	0.056	0.346	0.436	0.470
7	0.638	0.580	0.451	0.477	0.516	0.508	0.697	0.623	0.254	0.401
8	0.114	0.361	0.663	0.597	0.860	0.781	0.288	0.412	0.407	0.457
9	0.458	0.480	0.876	0.801	0.836	0.753	0.000	0.333	0.671	0.603
10	0.294	0.415	0.663	0.598	0.684	0.613	0.410	0.459	0.491	0.496
11	0.734	0.652	0.123	0.363	0.276	0.408	0.821	0.737	0.482	0.491
12	0.970	0.943	0.520	0.510	0.632	0.576	0.681	0.611	0.160	0.373
13	0.630	0.575	0.663	0.598	0.804	0.718	0.438	0.471	0.084	0.353
14	0.753	0.670	0.206	0.386	0.214	0.389	0.687	0.615	0.766	0.681
15	0.127	0.364	0.553	0.528	0.524	0.512	0.629	0.574	0.439	0.471
16	0.667	0.600	0.696	0.622	0.801	0.715	0.424	0.464	0.065	0.348
17	0.000	0.333	0.199	0.384	0.254	0.401	0.903	0.838	0.645	0.585
18	0.401	0.455	0.422	0.464	0.485	0.493	0.700	0.625	0.714	0.636
19	0.242	0.398	0.321	0.424	0.385	0.448	0.742	0.660	0.575	0.540
20	0.753	0.669	0.528	0.514	0.617	0.566	0.702	0.627	0.207	0.387
21	0.953	0.915	0.111	0.360	0.197	0.384	0.790	0.704	0.523	0.512
22	0.601	0.556	0.402	0.455	0.542	0.522	0.931	0.879	0.152	0.371
23	0.772	0.687	0.389	0.450	0.565	0.534	0.774	0.689	0.515	0.508
24	0.052	0.345	0.000	0.333	0.035	0.341	1.000	1.000	0.807	0.722
25	0.637	0.580	0.338	0.430	0.499	0.500	0.910	0.847	0.222	0.391
26	0.070	0.350	0.514	0.507	0.579	0.543	0.727	0.647	0.333	0.429
27	0.359	0.438	0.046	0.344	0.000	0.333	0.741	0.659	1.000	1.000

**Table 7:** Mean grey relational degree at each level for each factor in the automotive door TWB structure.

Factor		A	B	C	D	E	F	G	H	I	J	K	L
*d*_*sag*_	Level 1	0.6255	0.5452	0.6510	0.6444	0.6396	0.6449	0.6630	0.8952	0.6370	0.6346	0.6626	0.6482
	Level 2	0.6441	0.6442	0.6369	0.6406	0.6296	0.6403	0.6387	0.5810	0.6407	0.6436	0.6400	0.6342
	Level 3	0.6549	0.7351	0.6365	0.6395	0.6553	0.6393	0.6228	0.4483	0.6468	0.6463	0.6219	0.6420
	Δ	0.0293	0.1900	0.0145	0.0049	0.0257	0.0055	0.0402	0.4469	0.0098	0.0117	0.0407	0.0140
	Rank	5	2	7	12	6	11	4	1	10	9	3	8
*d*_*upper*_	Level 1	0.5183	0.6563	0.6680	0.6518	0.6551	0.6660	0.8448	0.6756	0.6649	0.6932	0.6830	0.6649
	Level 2	0.6794	0.6618	0.6629	0.6561	0.6687	0.6526	0.6210	0.6611	0.6543	0.6554	0.6597	0.6528
	Level 3	0.7888	0.6685	0.6557	0.6787	0.6627	0.6680	0.5208	0.6499	0.6674	0.6380	0.6438	0.6689
	Δ	0.2705	0.0121	0.0123	0.0269	0.0136	0.0154	0.3240	0.0257	0.0131	0.0552	0.0391	0.0161
	Rank	2	12	11	5	9	8	1	6	10	3	4	7
*d*_*lower*_	Level 1	0.4881	0.6040	0.6134	0.6193	0.5819	0.6035	0.7987	0.6362	0.6111	0.6085	0.6545	0.6149
	Level 2	0.6224	0.6109	0.6113	0.6062	0.6046	0.6105	0.5571	0.6044	0.6028	0.6118	0.6052	0.6062
	Level 3	0.7179	0.6135	0.6038	0.6029	0.6419	0.6144	0.4726	0.5879	0.6146	0.6081	0.5687	0.6073
	Δ	0.2298	0.0095	0.0096	0.0164	0.0600	0.0110	0.3262	0.0484	0.0118	0.0037	0.0859	0.0087
	Rank	2	10	9	6	4	8	1	5	7	12	3	11
*f*	Level 1	0.4370	0.5403	0.6285	0.5422	0.5884	0.5934	0.6781	0.5926	0.5520	0.5726	0.5305	0.6133
	Level 2	0.5668	0.5958	0.5570	0.5551	0.5661	0.5275	0.5643	0.5637	0.5761	0.5666	0.5939	0.5563
	Level 3	0.7049	0.5726	0.5232	0.6114	0.5542	0.5879	0.4663	0.5524	0.5806	0.5695	0.5843	0.5391
	Δ	0.2680	0.0554	0.0716	0.0693	0.0222	0.0659	0.1138	0.0289	0.0286	0.0061	0.0635	0.0570
	Rank	1	8	3	4	11	5	2	9	10	12	6	7
*Mass*	Level 1	0.6411	0.6477	0.6361	0.6409	0.6815	0.6105	0.4934	0.5126	0.6137	0.5727	0.4592	0.5977
	Level 2	0.6086	0.5950	0.5943	0.5924	0.5796	0.6080	0.6351	0.6306	0.5861	0.6195	0.6577	0.5967
	Level 3	0.5695	0.5765	0.5887	0.5860	0.5580	0.6008	0.6906	0.6759	0.6193	0.6270	0.7023	0.6248
	Δ	0.0715	0.0712	0.0474	0.0549	0.1235	0.0097	0.1972	0.1633	0.0332	0.0543	0.2431	0.0280
	Rank	5	6	9	7	4	12	2	3	10	8	1	11

**Table 8:** Comparison of door weight and dynamic performance before and after optimisation.

Parameter	Initial Design	Optimal Design	Variation
Mass, *M* (kg)	27.71	24.88	−10.21%
Natural frequency, *f* (Hz)	42.68	42.30	−0.89%
Vertical sag, *d*_*sag*_ (mm)	1.64	1.48	−9.76%
Upper lateral, *d*_*upper*_ (mm)	1.03	1.04	0.97%
Lower lateral, *d*_*lower*_ (mm)	2.21	2.39	8.14%

## Data Availability

Not applicable.
